# An Invitro Study to Evaluate the Retentive Properties of Metal Crowns on Various Surface Roughnesses of Abutments

**DOI:** 10.7759/cureus.67019

**Published:** 2024-08-16

**Authors:** Athira Krishna K, Pattathil Abdul Razak, Aparna Sooraj, Tessa Kuriachan, Lakshmi Radhakrishnan, Neethu Niduvote Poyil

**Affiliations:** 1 Department of Prosthodontics, Sree Anjaneya Dental College, Calicut, IND; 2 Department of Prosthodontics, MES Dental College, Perinthalmanna, IND; 3 Department of Prosthodontics, Implantree Dental and Aesthetic Centre, Kochi, IND; 4 Department of Prosthodontics, Kannur Dental College, Kannur, IND

**Keywords:** metal crowns, retention, restorative dentistry, abutments, surface roughness

## Abstract

Background

Restorative dentists frequently deal with the prosthesis coming loose after placing multiple crowns. The luting cement holds indirect restorations to the prepared tooth. However, the success of the restorations is impacted by mastication pressures and other undesired factors. Therefore, escape is required to increase the crown's life. Mechanical locking of the prepared tooth surface is one technique to address this issue, in addition to cement adherence, to extend the life of the restoration.

Aims and objective

The objective of the study was to evaluate the influence of surface roughness of prepared teeth on the retention of metal crowns.

Methodology

This in-vitro investigation was carried out on freshly extracted maxillary first premolars that were defect-free and had the same crown size. Using multiple grifts of varied coarseness, different surface roughness was created, allowing for the observation of an important factor like retention (black at 180-250 µm [micrometer], blue at 125-150 µm, green at 106-125 µm, red at 53-63 µm, yellow at 20-30 µm).

Results

IBM Corp. Released 2011. IBM SPSS Statistics for Windows, Version 20.0. Armonk, NY: IBM Corp. was used to perform the statistical analysis. Compounds were done before it began to guarantee that the study would have 80% power. There is a mean and a standard deviation for each quantitative variable. A one-way ANOVA was used for quantitative variables, and Tukey's post hoc analysis was conducted afterward. A probability value of less than 0.05 was used to determine statistical significance. According to the statistical findings, the prosthesis's retentive qualities improve as coarseness increases.

Conclusion

The resistance and retention form of the preparation is critical to the longevity of the prosthesis, based on the findings of the previously described study. Surface roughness, pins, slots, grooves, and other preparation modifications can enhance retention on the prepared tooth surface. The research findings indicate no need to polish the prepared tooth surface.

## Introduction

The need to maintain the dentition's functionality and aesthetics in all respects is becoming more widely recognized in our population. Among these, younger patients prefer fixed restorations over removable prostheses. A few years ago, most people in all social strata were unwilling to pay for dental care, even restoration and rehabilitation. As a result, they didn't expect their prosthesis to last as long as they do now that they understand how important it is to maintain dentition for proper functioning, better appearance, and increased confidence. When it comes to fixed prostheses, the patient always finds it bothersome when they come loose [1,2].

Dislodgement can occur for several reasons, including the prepared tooth's taper, finish lines, abutment surface area, crown size, prosthesis type (long or short span), patient characteristics, etc. [1]. It is crucial to choose the right degree of taper while preparing teeth. A taper that is too big won't be retentive, while one that is too little could result in undesired undercuts. It is advised that there be a 6-degree convergence between opposing walls. A tool with the desired taper held at a steady angulation should be used to prepare the tooth. Whenever feasible, the finish line should be positioned where the patient can maintain cleanliness and the dentist can finish the restoration margins.

By placing the finish lines in a way that keeps the cement from touching the oral fluids, the barrier they establish helps to stop microleakage, increasing the restoration's retention and lifespan. They also support the acrylic, porcelain, and metal used in restoration. Bevel shoulder, chamfer, knife-edge, and shoulder are the four fundamental varieties of finishing lines. Retention of the restoration depends on the length of the path of withdrawal, or more accurately, the surface area in sliding contact, if the restoration has a limited path of withdrawal. Hence, molar crowns of the same taper are more retentive than premolar crowns of the same taper, and crowns with long axial walls are more retentive than those with short axial walls. Long-span bridges not only put more strain on the periodontal ligament, but their larger spans are also less retentive because of their decreased rigidity [1-3].

Various researchers state that every bur imparts a different level of roughness to the prepared tooth. The smoothest surface is formed by yellow bur, while the highest degree of roughness is formed by black bur. The study aimed to hypothesize that mechanical interlocking combined with zinc phosphate cement adhesion may aid metal crown retention. Thus, the study's objective was to verify the importance of the surface roughness of the prepared tooth to aid in the retention of the prosthesis.

## Materials and methods

Study design and selection criteria

**Figure 1:**
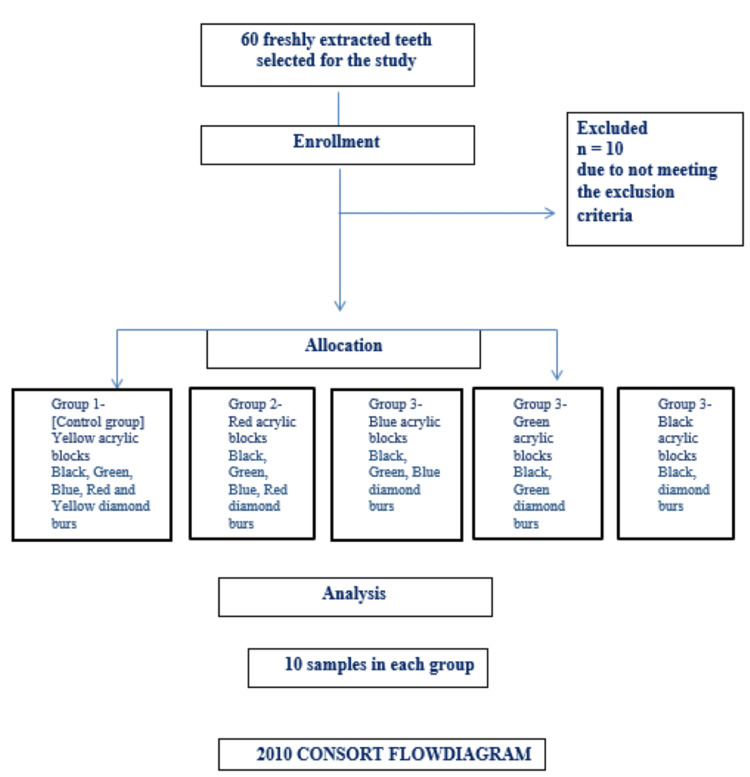
Flow chart of the study

**Figure 2:**
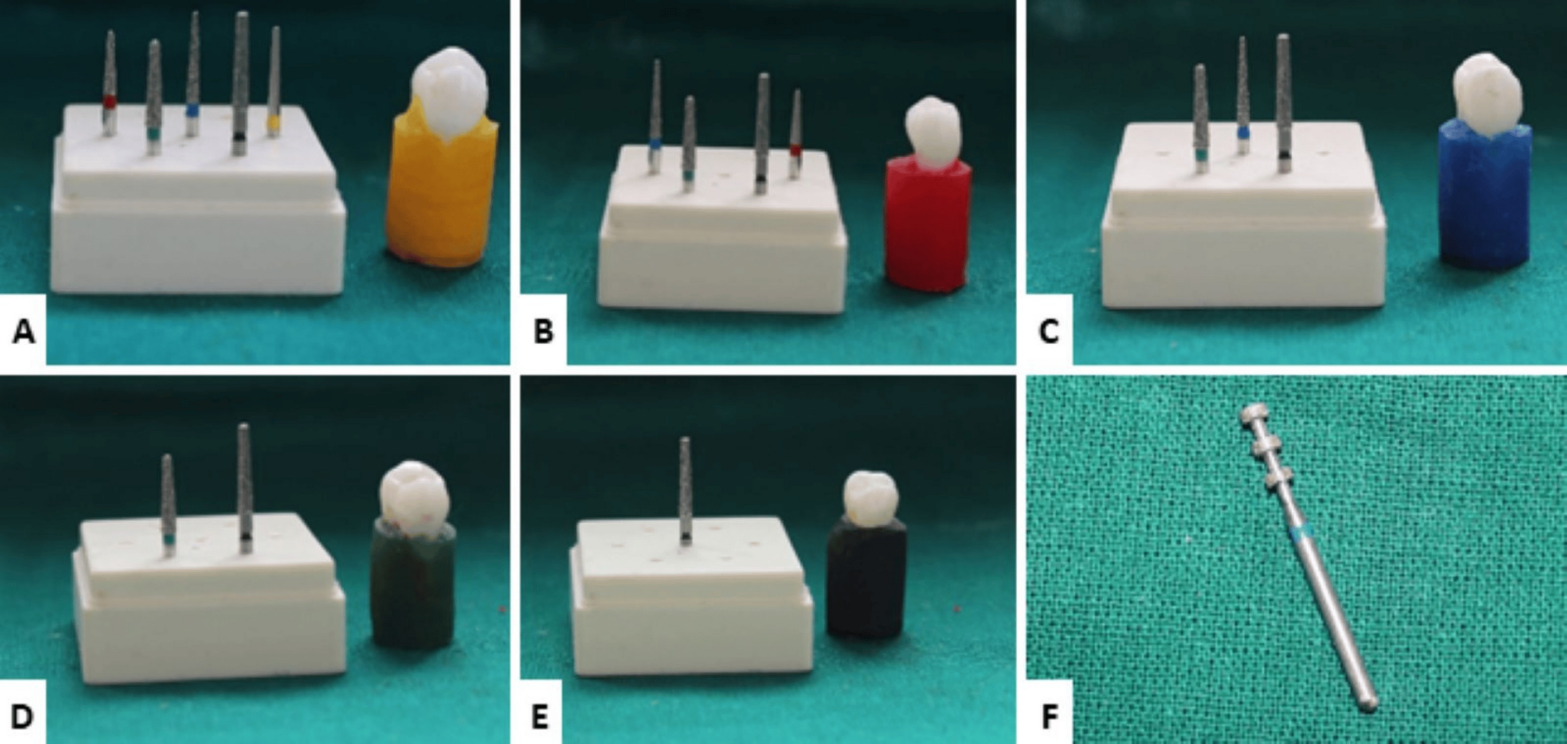
Color codlings of the acrylic blocks based on different groups and depth indicating bur Refer to Tables 1, 2 for details of the Groups shown in the panels in this Figure. A: Group 1, B: Group 2, C: Group 3, D: Group 4, E: Group 5, F: Depth indicating bur

This invitro study was approved by the institutional ethical committee (No. IEC/MES/67/2019, dated November 20, 2019). The study analyzed and compared the retentive property after surface roughness was made using different burs. Study samples include 50 freshly extracted maxillary first premolars without any defects, with the same crown size, and were randomly used in the study. Samples were randomly divided into five groups, with 10 samples in each group. The specimen's roots were embedded in acrylic blocks with a notch [2]. The flow chart of the study is explained in Figure 1. The surface roughness of the preparation was the only variable that was altered in this investigation [3]. A high-speed air-turbine rotary handpiece with a coolant water spray of 25 mL/min was fixed onto the device, which operated at 300,000 rpm. The following diamond burs are used in the following order in the traditional method of tooth preparation: black, green, blue, red, and yellow. In the first group, which serves as our control, all of the burs (Mani Diamond Bur Manufacturing Inc., New Delhi, India) were employed in the following order: black, green, blue, red, and yellow. As a result, each group's bur count was decreased to enhance surface roughness [2]. Yellow was left out of group two. Red and yellow were left out of group three. Green and black were used in group four. Only black was utilized in group five (Table 1). Burs were cleaned with a toothbrush following each preparation step and then put in an ultrasonic cleaner (Bandelin Sonorex RK 102 P; Bandelin Electronic, Berlin, Germany) for five minutes. The various colors of the acrylic blocks in Tables 1, 2, and Figure 2 indicate the following groupings.

**Table 1:** Color coding of the acrylic blocks based on different groups

Group Number	Color code
Group 1	Yellow
Group2	Red
Group3	Blue
Group4	Green
Group5	Black

**Table 2:** Categories of specimens based on the burs used

Group	Burs Used
Group 1 [Control Group]	Five burs are used: Super coarse -Black [BL], Coarse bur - Green [G], Moderate bur - Blue [B], Fine bur -Red [R], Ultra-fine bur-Yellow [Y],
Group 2	Four burs are used: Super coarse – Black [BL], Coarse bur - Green [G], Moderate bur - Blue [B], Fine bur - Red [R]
Group 3	Three burs are used: Super coarse -Black [BL], Coarse bur - Green [G], Moderate bur-Blue [B]
Group 4	Two burs are used; Super: coarse – Black [BL], Coarse bur - Green [G]
Group 5	One bur is used: Super coarse – Black [BL]

Procedure

**Figure 3:**
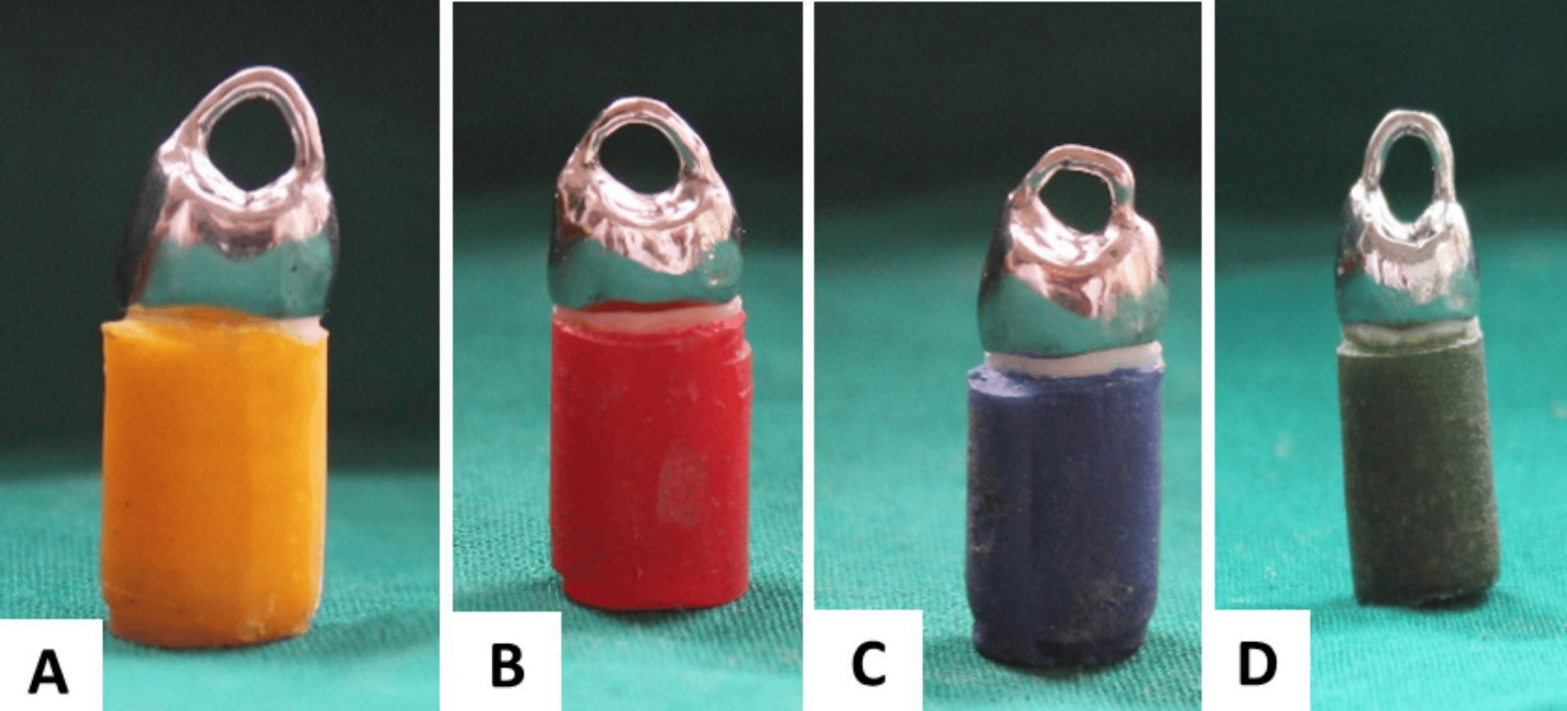
Specimens with luted metal crowns Each panel shows the different color-coded specimens with luted metal crowns

First, all the specimens were kept in artificial saliva before being used for the procedure [4]. Each bur creates a different surface roughness: super-coarse with a surface roughness of 181 µm, coarse with 151 µm, medium with 107-126 µm, and finally fine with 40 µm. The specimen height in each group was limited to 3 mm, with a 6-degree convergence angle and a chamfered finish line [5]. The convergence angle (CA) of tooth preparation is the measurement of the combined taper of opposing axial walls, and it is the angle created by the intersection of the mesial and distal axial wall tapers. It is well acknowledged that a restoration would retain its integrity better and last longer if the convergence angle was lower [6]. According to theoretical recommendations, tooth preparation walls should be as nearly parallel as feasible but should yet have a small taper, ideally between 4° and 6°, with a recommended range of 3° to 14° being seen as appropriate. The crown's occlusal surface was smoothed over, and the preparation's length was preserved by using a depth-indicating bur. The preparation was brought to a consistent convergence using a tapered fissure bur. The airotor will be fixed to the milling machine [NSK Pana Air Handpiece FX TB2]. To ensure uniformity in the preparation, the same dentist completed each step of the process. There was just one grit used per specimen [3]. The specimens were transported to the lab to make metal crowns, as depicted in Figure 3. Nickel-chromium alloys (Wiron Light, ref. 50270, lot 73784, BEGO Bremer Goldschlägerei Wilh. Herbst GmbH & Co. KG., Bremen, Germany) have been used to fabricate metal crowns.

**Figure 4:**
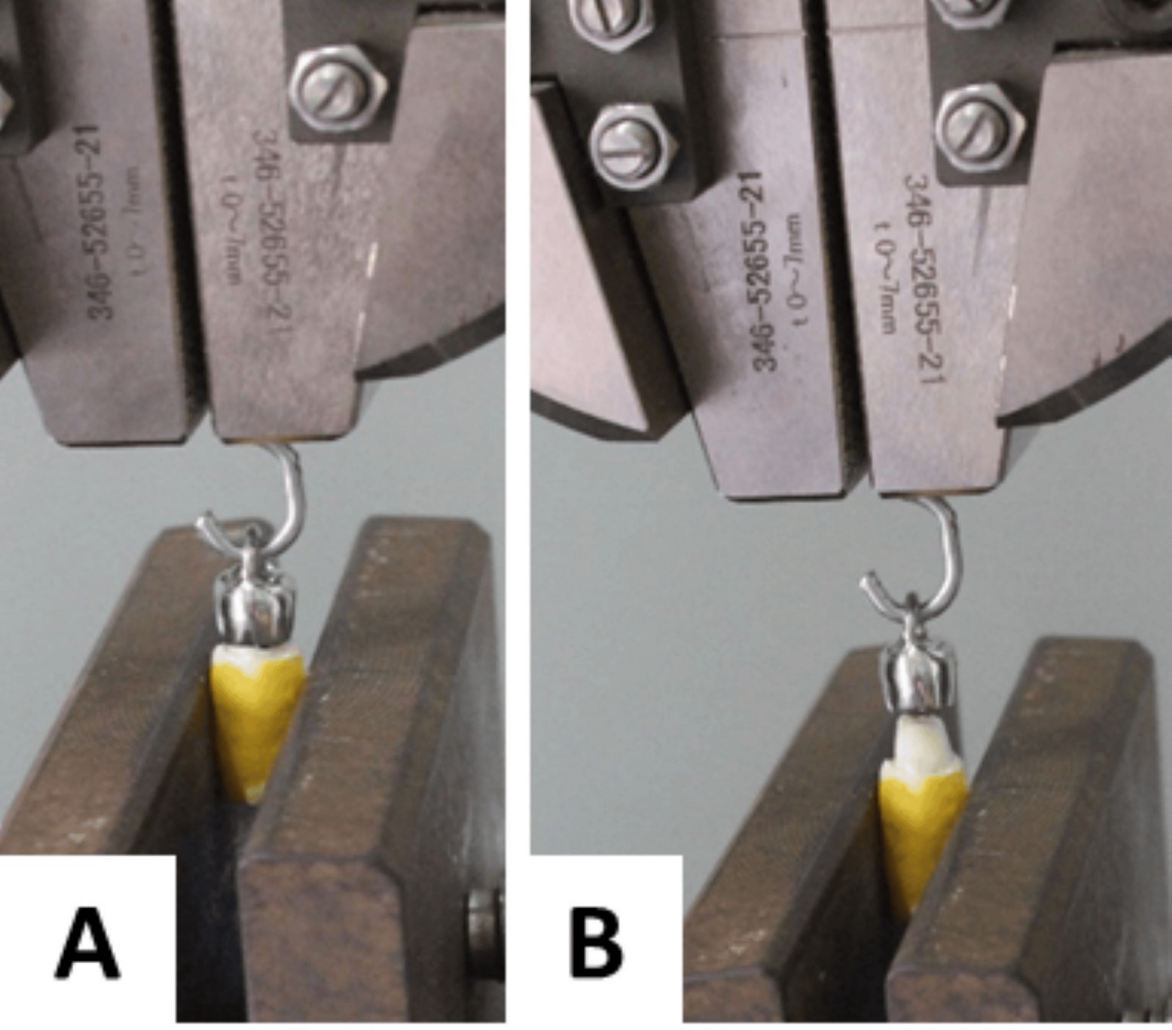
Universal testing machine A: Specimen attached to UTM, B: Image showing dislodged crown

A loop or ring construction was created to make it easier to attach the crown to the end of the universal testing machine (UTM) (AG-X plus 10kN, manufactured by Shimadzu Autograph, Kyoto, Japan) [7]. TRAPEZIUM X data processing software was used for the data reading. Zinc phosphate cement (manufactured by Dentsply DeTrey GmbH, Germany) was used to bind these crowns to the prepared teeth, as shown in Figure 2. Using universal load testing equipment, specimens were transported to NIT Calicut, Kerala, to evaluate the tensile strength needed to remove the crown from different surface roughness preparations on the tooth surface [8]. The same is depicted in Figure 4. 

Statistical analysis

IBM Corp. Released 2011. IBM SPSS Statistics for Windows, Version 20.0. Armonk, NY: IBM Corp. was used for all statistical operations. Every quantitative variable has a mean and a standard deviation. For quantitative variables, a one-way ANOVA was utilized, followed by Tukey's post hoc analysis. Statistical significance was defined as a probability value of less than 0.05. The minimum sample size required to produce a statistical power of at least 80% with an alpha of 5% and a medium effect size (d=0.45) for a one-way analysis of variance (ANOVA) was 60, according to power analysis. G* Power version 3.1.9.7 (2020) software (Heinrich Heine University Düsseldorf, Düsseldorf, Germany) was used to estimate the sample size. Ten were excluded for not meeting the exclusion criteria, thus making the total sample 50.

## Results

**Figure 5:**
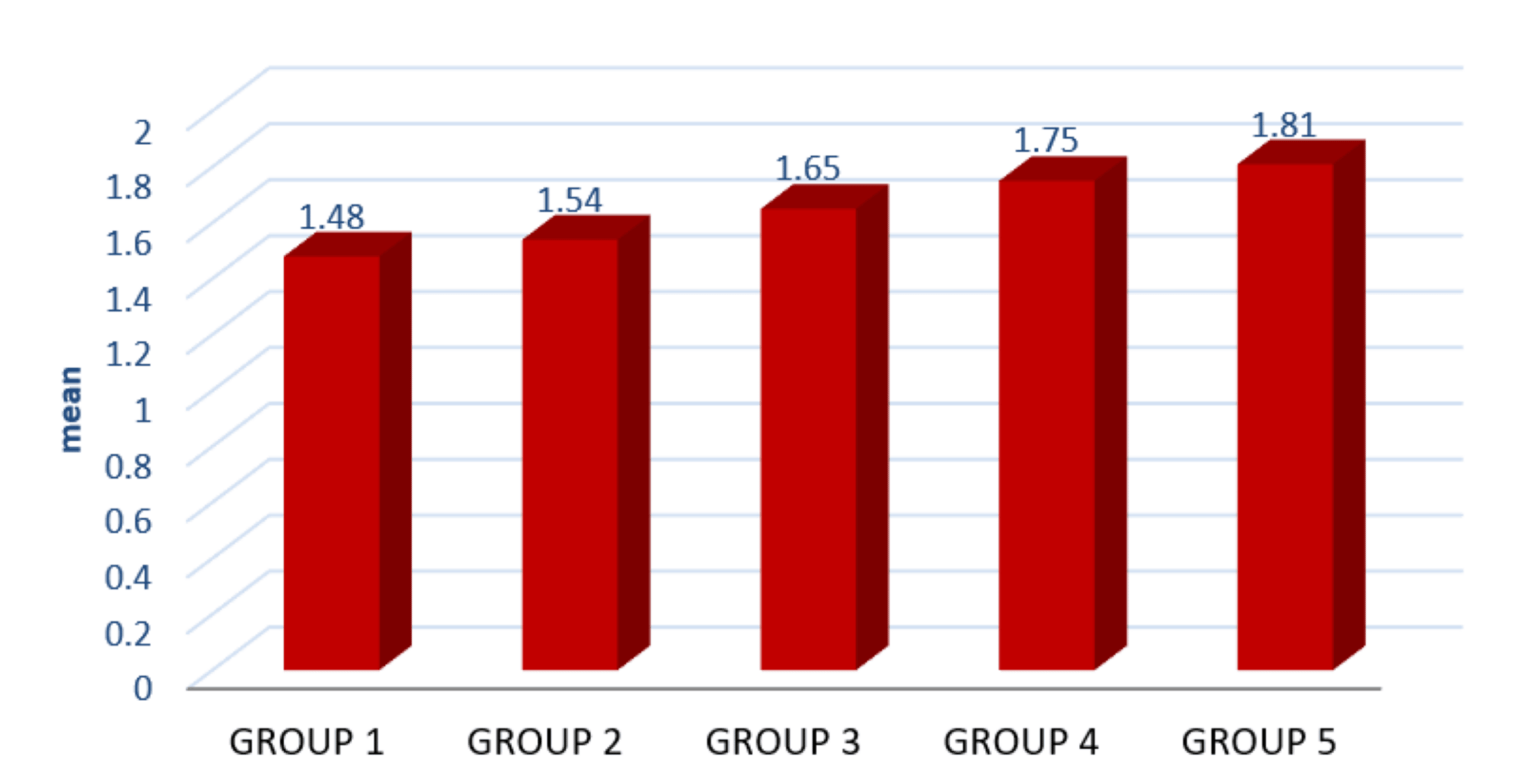
Graphical representation of the retentive properties of metal crowns on various surface roughnesses of abutments Refer to Tables 1, 2 for details of the Groups

The study comprised five groups, with 10 specimens in each group. Table 3 and Figure 5 compare the retentive properties of metal crowns on various surface roughnesses of abutments. Statistically significant differences were observed in five groups (p-value <0.001**).

**Table 3:** Comparison of the retentive properties of metal crowns on various surface roughnesses of abutments p-value <0.05 was considered statistically significant; ** p-value <0.001  was considered statistically significant Refer to Tables 1, 2 for details of the Groups

Name of Group	N	Mean ± Standard Deviation	p-value
Group 1	10	1.48 ± 0.04	<0.001**
Group 2	10	1.54 ± 0.04	
Group 3	10	1.65 ± 0.05	
Group 4	10	1.75 ± 0.05	
Group 5	10	1.81 ± 0.08	

Table 4 shows the multiple comparisons of the retentive properties of metal crowns on various surface roughnesses of abutments. Significant differences were noted when group 1 was compared with groups 3, 4, and 5. When group 2 was compared with others, significant differences were observed in groups 3, 4, and 5. On comparing Group 3 with others, statistically significant differences were found between the other groups. Groups 1, 2, and 3 showed statistically significant differences from Group 4. Group 5 showed significant differences with groups 1, 2, and 3 on intergroup comparison. 

**Table 4:** Multiple comparisons of the retentive properties of metal crowns on various surface roughnesses of abutments p-value <0.05 was considered statistically significant; ** p-value <0.001 was considered statistically significant; Refer to Tables 1, 2 for details of the Groups

(I) VAR00001	(J) VAR00001	Mean Difference (I-J)	Standard Error	p-value	95% Confidence Interval	
					Lower Bound	Upper Bound
Group 1	Group 2	-0.05560	0.02497	00.189	-0.1265	0.0153
	Group 3	-0.17170^*^	0.02497	00.001*	-0.2426	-0.1008
	Group 4	-0.26390^*^	0.02497	00.000**	-0.3348	-0.1930
	Group 5	-0.33220^*^	0.02497	00.000**	-0.4031	-0.2613
Group 2	Group 1	0.05560	0.02497	00.189	-0.0153	0.1265
	Group 3	-0.11610^*^	0.02497	00.000**	-0.1870	-0.0452
	Group 4	-0.20830^*^	0.02497	00.000**	-0.2792	-0.1374
	Group 5	-0.27660^*^	0.02497	00.000**	-0.3475	-0.2057
Group 3	Group 1	0.17170^*^	0.02497	00.000**	0.1008	0.2426
	Group 2	0.11610^*^	0.02497	00.000**	0.0452	0.1870
	Group 4	-0.09220^*^	0.02497	00.005*	-0.1631	-0.0213
	Group 5	-0.16050^*^	0.02497	00.000**	-0.2314	-0.0896
Group 4	Group 1	0.26390^*^	0.02497	00.000**	0.1930	0.3348
	Group 2	0.20830^*^	0.02497	00.000**	0.1374	0.2792
	Group 3	0.09220^*^	0.02497	00.005*	0.0213	0.1631
	Group 5	-0.06830	0.02497	00.064	-0.1392	0.0026
Group 5	Group 1	0.33220^*^	0.02497	00.000**	0.2613	0.4031
	Group 2	0.27660^*^	0.02497	00.000**	0.2057	0.3475
	Group 3	0.16050^*^	0.02497	00.000**	0.0896	0.2314
	Group 4	0.06830	0.02497	00.064	-0.0026	0.1392

## Discussion

This in-vitro study aims to evaluate the impact of varying surface roughness on the retentive properties of metal crowns. By examining five distinct groups, each comprising 10 specimens, the research seeks to understand how different surface textures influence crown retention. The discussion reveals significant findings, as illustrated in Table 3 and Figure 5, showing statistically significant differences across the groups (p < 0.001). Table 4 further details these differences, indicating that group 1's retentive properties differ significantly when compared to groups 3, 4, and 5. Similarly, group 2 exhibits significant differences with groups 3, 4, and 5. Notably, group 3's retentive properties also show significant variance when compared to the other groups. Furthermore, groups 1, 2, and 3 differ markedly from group 4, and group 5 shows significant differences when compared to groups 1, 2, and 3. These findings underscore the impact of abutment surface roughness on the retention of metal crowns and highlight the nuanced interplay between different levels of surface roughness.

Natural teeth mounted on color-coded acrylic blocks were used for the investigation. Premolars that had just been extracted served as the specimens. Fifty specimens were used for the investigation; they were divided into five groups and mounted on acrylic blocks that were color-coded. Different color codes are provided for the acrylic blocks. The yellow acrylic block falls under Group 1, red in Group 2, blue in Group 3, green under Group 4, and Group 5 contains black acrylic block. The groups were given a color code based on the diamond grits used. Group 1 served as the control group.

Debonding of the cemented crown, mostly caused by the weak adhesion between the metal and tooth surface, is the primary factor affecting treatment outcomes. This significant problem was mitigated by the casting surface being etched chemically or electrolytically, adding mechanical retention, and using aluminum oxide particles for air abrasion. Even with chemically active resin cements, robust repair adhesion is still unsatisfactory. Patients would still frequently complain about their crowns coming loose and causing problems for the dentist [6]. Metal crown longevity has increased due to preparation adjustments such as retentive grooves, slots, pins, and other biomechanical features, as shown in multiple investigations [6-10]. Retention and resistance forms derived from the concepts of tooth preparation are the decisive factors in better clinical retention of restorations. Retentive features increase prosthesis retention but also decrease prepared tooth surface area, which is important for bonding and thus impacts prosthesis retention [8]. 

Increasing surface roughness can be a way to improve prosthesis retention, as it has been demonstrated that retention rises as contact surface area increases. There are, however, very few small studies comparing grits, the surface roughness they produce, and how they affect prosthesis retention. In their investigation, Garber et al. [9] demonstrated that cross-cut carbide burs improved retention by roughly 46% to 55%. This study recommends that the preparation's surface roughness be considered to increase prosthesis retention. Using rotary tools to roughen the preparation surface can improve retention and the cement teeth interlocking process. This reduces the need for additional retentive features like slots and pins [7-9].

The ideal restoration should meet mechanical, biological, and aesthetic criteria. The preservation of the tooth of interest, gingiva, neighboring teeth, and other living tissue are all considered when preparing for biological reasons [9,10]. A diamond is precisely placed along the desired withdrawal path to prepare the tooth mechanically. Preparation thickness should be limited to half of the bur; excessive reduction results in retention loss.

Three categories apply to mechanical considerations: retention form, resistance form, and keeping the repair from deforming. The amount of preparation necessary to keep the restoration from being moved by such forces parallel to the placement path is known as the retention form. Various factors, such as the magnitude of dislodging force, the geometry of tooth preparation, and surface roughness, improve the retention of FPD [3]. Less magnitude of dislodging force tends to remove restorations than by forces that are typically to tilt them. Eating sticky food causes the greatest amount of removal.

The geometry of the tooth preparation is another important factor that determines retention. An adequate amount of taper provided during the tooth preparation is a requirement to enhance retention [11]. The taper of a crown preparation is the point at which two opposing, facing external walls converge when viewed in a certain plane. Maximum retention is attained when the preparation's walls are parallel. When two axial walls face in different directions, undercuts may occur. Clinically, a small taper for convergence is appropriate. Retention falls off as taper increases. The increased surface area provides more retention. Molar crowns are more retentive than premolars because retention increases with surface area. Stress concentration also has to be kept in mind. Sharp occlusoaxial line angles should not be sharp to limit stress and partially lessen restoration failure.

Retention is also increased by adding grooves or boxes with a confined placement path, limiting the taper of the preparation, and increasing the surface area of the axial walls [12]. The roughness of the fitting surface of the restoration: Surface roughness improves prosthesis retention by increasing surface area. The spiral or helix pattern created on a surface by the "tool marks" left by the cutting instrument is known as true surface roughness (Ra). This contrasts with the "straightness" resulting from form defects, such as those made during free-hand surface cutting, and the "waviness" brought on by vibration. Longer wavelengths are produced by both waviness and straightness than by roughness. The surface profile comprises the three criteria of roughness, waviness, and straightness. Because the three are wavelengths with varying intervals, a filter can analyze surface roughness at a high frequency. The American National Standard on Surface Texture (ANSI B46.1-1978) states that the surface measurement must meet two criteria [13].

Either the core or building material and the casting alloy will impact retention. It is stated that the greater the adhesion between the luting agents and the alloy, the more retentive it is. As a result, base metal alloys containing nickel, cobalt, and chromium are better at retaining information than less reactive metals with high gold content [13]. The type of luting agent selected influences the retention of a cemented repair. The five types of luting chemicals that are most frequently utilized are resin-bonded cement, zinc oxide eugenol, glass ionomer, zinc phosphate, and zinc polycarboxylate. The primary mechanism for retaining restorations has been the mechanical interlocking of cement into imperfections on the interior surface of the prepared tooth and the produced restoration. In addition to mechanical interlocking, polycarboxylate, and glass ionomer cement bind directly to calcify tissues by chemical attraction to calcium ions [14]. Because it may lessen microleakage between the restoration and the tooth, true adhesion between cement and tooth is preferred [15].

It has been demonstrated that retention failure occurs if the internal surface of the restoration is extremely smooth [16]. Therefore, it is advised to use 50μm alumina to air abrade the casting's interior surface. More reactive alloys, such as those containing nickel, cobalt, and chromium, have been shown to retain information better than less reactive metals with high gold content. The influence of a thicker cement film on restoration retention has been the subject of conflicting research. However, it has been demonstrated that a consistent cement thickness between the restoration and the tooth offers greater retention than a non-uniform thickness. A film thickness of 2.5 5μm or less has been recommended for satisfactory restoration [15,16].

Limitations

The study had several limitations due to its in vitro design. Using a homogeneous sample of maxillary first premolars may not reflect real-world variability in tooth characteristics, affecting generalizability. The controlled environment also fails to simulate biological influences like saliva, impacting material performance. The experiment’s focus on immediate outcomes using a fixed sequence of burs may not consider the diverse techniques used by clinicians or long-term durability, which is crucial for clinical relevance. Additionally, the unspecified sample size could undermine the statistical robustness of the findings.

## Conclusions

According to the results of the aforementioned study, the resistance and retention form of the preparation is crucial to the prosthesis' durability. Pins, slots, grooves, and other preparation adjustments, as well as surface roughness, all help to improve retention on the prepared tooth surface. Additionally, it was shown that polishing is not an essential step in tooth preparation. According to the results, the tooth surface roughness was highest in samples prepared with ultra-coarse grain, and the retention value was highest in samples tested with a universal testing machine.
